# N-terminal and C-terminal heparin-binding domain polypeptides derived from fibronectin reduce adhesion and invasion of liver cancer cells

**DOI:** 10.1186/1471-2407-10-552

**Published:** 2010-10-13

**Authors:** Nan-Hong Tang, Yan-Lin Chen, Xiao-Qian Wang, Xiu-Jin Li, Yong Wu, Qi-Lian Zou, Yuan-Zhong Chen

**Affiliations:** 1 Fujian Institute of Hepatobiliary Surgery, Union Hospital, Fujian Medical University, Fuzhou, China; 2 Fujian Institute of Hematology, Union Hospital, Fujian Medical University, Fuzhou, China; 3 Division of Cell Biology and Genetics, Fujian Medical University, Fuzhou, China

## Abstract

**Background:**

Fibronectin (FN) is known to be a large multifunction glycoprotein with binding sites for many substances, including N-terminal and C-terminal heparin-binding domains. We investigated the effects of highly purified rhFNHN29 and rhFNHC36 polypeptides originally cloned from the two heparin-binding domains on the adhesion and invasion of highly metastatic human hepatocellular carcinoma cells (MHCC97H) and analyzed the underlying mechanism involved.

**Methods:**

The MHCC97H cells that adhered to FN in the presence of various concentrations of rhFNHN29 and rhFNHC36 polypeptides were stained with crystal violet and measured, and the effects of rhFNHN29 and rhFNHC36 on the invasion of the MHCC97H cells were then detected using the Matrigel invasion assay as well as a lung-metastasis mouse model. The expression level of integrins and focal adhesion kinase (FAK) phosphotyrosyl protein was examined by Western blot, and the activity of matrix metalloproteinases (MMPs) and activator protein 1 (AP-1) was analyzed by gelatin zymography and the electrophoretic mobility band-shift assay (EMSA), respectively.

**Results:**

Both of the polypeptides rhFNHN29 and rhFNHC36 inhibited adhesion and invasion of MHCC97H cells; however, rhFNHC36 exhibited inhibition at a lower dose than rhFNHN29. These inhibitory effects were mediated by integrin αvβ3 and reversed by a protein tyrosine phosphatase inhibitor. Polypeptides rhFNHN29 and rhFNHC36 abrogated the tyrosine phosphorylation of focal adhesion kinase (p-FAK) and activation of activator protein 1 (AP-1), resulting in the decrease of integrin αv, β3 and β1 expression as well as the reduction of MMP-9 activity.

**Conclusions:**

Polypeptides rhFNHN29 and rhFNHC36 could potentially be applicable to human liver cancer as anti-adhesive and anti-invasive agents.

## Background

Invasion and metastasis are important biological characteristics of malignant tumors. Metastatic formation requires specific cell-to-cell and cell-to-extracellular matrix (ECM) interactions mediated by integrins [1], cadherins [2], selectins [3], etc. In particular, integrin-mediated adhesion of tumor cells to ECM proteins and cell surface components is considered a crucial event in metastasis. Accordingly, the prevention of tumor cell adhesion to ECM proteins has been an area of interest as a potential target for therapeutic intervention [4-6].

Fibronectin (FN) is a type of adhesive-attraction glycoprotein. Previous studies have shown that the anchoring of FN to ECM *in vitro *plays an important role in cancer cell metastasis [7]. Moreover, the study of FN expression on cancer cells has determined that decreased FN expression is closely associated with cancer growth and metastasis [8-10], which illustrates that an increase of FN expression in cancer cells may conversely facilitate the reduction of cancer cell metastasis, implying that FN may have the potential for a significant clinical application. Interestingly, our *in vitro *intervention experiments showed that free FN could inhibit the adhesion and metastasis of hepatocellular carcinoma cells. It is possible that the exposed cell binding site may not be the same in FN between free-status and anchoring-status. Therefore, the free FN has potential value for a therapeutic application. However, obstacles exist for the clinical application of FN, including deficiency of blood plasma, danger of blood-infection disease, and difficulty in engineering the synthesis of the whole-molecule FN with a molecular weight as large as 420 kDa. Fortunately, FN contains several active sites that serve as scaffoldings for cell anchorage [11], known as the heparin-binding domains, collagen-binding domain, fibrin-binding domain and cell-binding domain, respectively. These domains are involved in a diverse array of cell functions including adhesion, migration, differentiation, apoptosis, morphous change, haemostasis and reparation of damage, etc. Therefore, the replacement of FN with a polypeptide derived from a FN functional domain is considered to be a more feasible method for cancer treatment. Several reports have highlighted this type of application; for example, it has been found that arginine-glycine-aspartic acid (RGD) integrin-binding motif from FN containing the adhesion recognition signal Arg-Gly-Asp partially inhibit intrahepatic metastasis of murine hepatocellular carcinoma (HCC) [12]. The CH50 polypeptide that contains Cell I and Hep II dual domain fragment of FN has been shown to play a role involving inhibition of tumor growth, invasion and angiogenesis [13]. The FNIII14 peptide also effectively inhibited the adhesion and metastasis of lymphoma cells [14].

Heparin-binding domains are important molecular structures of FN: one (237 mer) at the N-terminal contains five I-type homologous structures [15], and another (272 mer) at the C-terminal contains three type III homologous structures [16]. In previous studies we obtained purified recombinant N-terminal and C-terminal heparin-binding domain polypeptides of FN (rhFNHN29 and rhFNHC36) using genetic engineering, further testing their characteristics with heparin-binding activity measurements [17]. However, the function of these fragments concerning cancer therapy was still unknown. In this study we investigated the effects of rhFNHN29 and rhFNHC36 on the adhesion and invasion of highly metastatic human HCC cells (MHCC97H) and analyzed the underlying mechanism.

## Methods

### Reagents, Cell Culture, and Animal Model

The reagents for the production of rhFNHN29 and rhFNHC36 were purchased: restriction enzyme *Xho*I, *EcoR*I, *BamH*I, *Hind*III, Bgl II and T4 ligase from Promega Co.; vector pPIC9K, pGEM-T, pAo815SM and GS115 yeast cells from Invitrogen Co.; purification column S-100, SP and HiTrap Heparin HP column from GE Co. BSA, type I collagen, type IV collagen and FN were purchased from Sigma Co.; and anti-FN pAb, anti-integrin αv (P2W7), β3 (BV4), β1 (8A2) mAb and anti-p-FAK mAb were purchased from Santa Cruz Biotechnology, Inc. Liver carcinoma cell lines (MHCC97H and MHCC97L) were purchased from Liver Cancer Institute of Fudan University (Shanghai, China) and maintained in DMEM medium supplemented with 10% fetal bovine serum (GIBCO) in a 5.0% CO_2 _incubator at 37°C [18]. Huh-7, HepG2, BEL7404 and SMMC7721 cells were maintained in our laboratory. Male BALB/c nude mice at the age of 5 to 6 weeks were purchased from the Chinese Academy of Sciences (Shanghai) and were given humane care according to the criteria outlined in the "Guide for the Care and Use of Laboratory Animals" prepared by the National Academy of Sciences and published by the National Institutes of Health (NIH publication 86-23 revised 1985). The experimental protocol was approved by the Medical Experimental Animal Care Committee of Fujian Medical University.

### Production of rhFNHN29 and rhFNHC36

According to the structural features of yeast expression vector pGEM-T, PCR primers to clone the rhFNHN29 and rhFNHC36 genes were designed and the sequences of these primers are as follows:

5'-ATGCTC^↓^TCGAGAAAAGAGAGGCTGAAGCTAGTCAAAGCAAGCCCGGTTGTTA-3'(rhFNHN29-F), 5'-ACGTAG^↓^AATTCTCCGCTCGATGTGGTCTGCA-3'(rhFNHN29-R), 5'-CGG^↓^GATCCGCTATTCCTGCACCAACTGAC-3'(rhFNHC36-F) and 5'-CCCA^↓^AGCTTCTCGTCTGTCTTTTTCCTTCC-3'(rhFNHC36-R), which contain *XhoI*/*EcoR*I and *BamH*I/*Hind*III sites (underlined), respectively.

The rhFNHN29 gene was then cloned into the integrated yeast expression vector pPIC9K through the transition of the pGEM-T and pAo815SM vector. The recombinant plasmid of pPIC9K-FNHN29 was confirmed by restriction analysis and sequenced upon transfection of competent DH5a *E.coli *cells and selection of resistant clones. The pPIC9K-FNHN29 plasmid was extracted, digested by BglII enzyme, and transferred into the GS115 yeast cells. The resistant clone with high expression was cultured for amplification. The fermentation liquid was precipitated using 80% ammonium sulfate, and then the dissolved sediments were purified using an S-100 column and an SP column. The UV280 waveform of the purified liquid was monitored by HPLC. The collected peak waveform sample was detected by SDS-PAGE electrophoresis and western blot analysis for FN antigenicity. The yeast fermentation supernatant of rhFNHN29 was diluted by 5 mM PBS with an adjusted 7.0 pH value and conductivity less than 4.0 and then eluted on a HiTrap Heparin HP column. HPLC was used to monitor the UV280 waveform of the purified liquid. The collected peak waveform sample was measured by mass spectrometry to determine the molecular weight. The construction and identification procedure for the recombinant yeast expression vector of rhFNHC36 was identical to the procedure above.

### Semiquantitative RT-PCR Analysis

RNA was isolated from the cell lines with Trizol (Invitrogen, USA) and was then reverse-transcribed. Fragments of integrin α4 (296 bp), α5 (362 bp), αv (236 bp), β1 (407 bp) and β3 (327 bp), and MMP-1 (428 bp), MMP-2 (390 bp), MMP-9 (215 bp) and β-actin (500 bp) cDNA were amplified by PCR. The primer sequences were as follows: integrin α4, sense 5'-CAACACGCTGTTCGGCTAC-3' and antisense (AS) 5'-TATGCCCACAAGTCACGATG-3'; integrin α5, sense 5'-CCGAATTCTGGAGTATGCAC-3' and AS 5'-TGGTGACATAGCCGTAAGTG-3'; integrin αv, sense 5'-GGAGCAATTCGACGAGCACT-3' and AS 5'-GCAGGCGTGAACTGGTTAAG-3'; integrin β1, sense 5'-TGAAGGGCGTGTTGGTAGAC-3' and AS 5'-GCCCTTGAAACTTCGGATCT-3'; integrin β3, sense 5'-CACCAGTAACCTGCGGATTG-3' and AS 5'-GTCTTGGCATCAGTGGTAAAC-3'; MMP-1, sense 5'-CTGAAGGTGATGAAGCAGCC-3' and AS 5'-AGTCCAAGAGAATGGCCGAG-3'; MMP-2, sense 5'-GCGACAAGAAGTATGGCTTC-3' and AS 5'-TGCCAAGGTCAATGTCAGGA-3'; MMP-9, sense 5'-AGTTCCCGGAGTGAGTTGAA-3' and AS 5'-CTCCACTCCTCCCTTTCCTC-3'; β-actin sense, 5'-ATGTCACGCACGATTTCCCGC-3' and AS 5'-GGCATGGGTCAGAAGGATTCC-3'.

### Cell Adhesion Assay

Part 1: The MHCC97H cell suspensions (2×10^5^/100 μl) with or without Mn^2+ ^(0.1 mM) were added to 96-well plates coated with either BSA, type I collagen, type IV collagen, or FN at 20 μg/ml. After the above incubation at 37°C for 1 h, cells were fixed with formalin, and separated cells were washed off with PBS [19]. Cells that adhered to the substrate were stained with crystal violet. Dye bound to adhered cells was solubilized with 0.1% SDS, and absorbance at 590 nm was measured. The data represent the mean value of three determinations. Part 2: The MHCC97H cell suspensions (2 × 10^5^/100 μl) containing Mn^2+ ^(0.1 mM) were pretreated with function-blocking mAb to αv (P2W7), β3 (BV4) or β1 (8A2) and control IgG at different concentrations (6, 12, 25 and 50 μg/ml), respectively, and were then seeded in 96-well plates coated with FN (20 μg/ml). The following procedures were identical to Part 1 above. Part 3: The MHCC97H cell suspensions (2 × 10^5^/100 μl) containing Mn^2+ ^(0.1 mM) were pretreated with or without 25 μg/ml mAb to αv (P2W7), β3 (BV4) or β1 (8A2) and control IgG, respectively, and were then seeded in 96-well plates coated with FN (20 μg/ml) or poly-L-Lys (20 μg/ml) in the presence or absence of FN, rhFNHN29 and rhFNHC36 at three different concentrations (50, 100 and 200 μg/ml). The procedure that follows is identical to Part1 above.

### Matrigel Invasion Assay

Transwell culture inserts with an 8-mm pore-size PET membrane precoated with 20 μg Matrigel (BD, USA) were transferred to a new 24-well plate. The bottom chambers were coated with 10 μl FN (0.5 mg/ml) and air-dried. The MHCC97H cell suspension (5 × 10^4^/100 μl) containing Mn^2+ ^(0.1 mM) was then treated with or without FN, rhFNHN29 and rhFNHC36 (50, 100 and 200 μg/ml) at 37°C for 1 h, and control IgG, or anti-αv (P2W7), -β3 (BV4) or -β1 (8A2) (25 μg/ml) was then added to the top chambers, respectively. After 48 h incubation, the inserts were dipped into a fixing solution (containing 95% ethanol and 5% acetic acid) for 30 min, and the non-invading cells were removed from the upper surface of the membrane by scrubbing with a cotton swab. The cells on the lower surface of the membrane were stained with H&E. Cells from five different fields were counted under an inverted microscope at 100-fold magnification and the mean was calculated.

### Experiments of Lung Metastasis

1 × 10^7 ^cells in a 0.2 ml volume of normal saline (NS) were inoculated subcutaneously into the right flank of nude mice. Seven days later, a tumor (approximately 5 mm in diameter) appeared at the cell inoculation site in each nude mouse model. 32 of the tumor-bearing nude mice were divided randomly into 4 groups (n = 8 per group), and then injected with NS (100 μl each time), FN, rhFNHN29 and rhFNHC36 (200 μg in 100 μl NS each time), respectively, at the tumor cell inoculation site for 7 consecutive days. At the end of 6 wk, all of the mice were sacrificed, and the lungs were excised, fixed in formalin, imbedded in paraffin, and cut into 4- μm consecutive sections and stained with H&E. Each metastatic focus in the lung was identified as it appeared at the same site on consecutive sections. Finally, all determined foci were counted to evaluate metastasis histologically [20,21]. Three pathologists performed the counting independently and in a blinded manner.

### MMP Activity

The MHCC97H cell (1.2 × 10^6^) suspensions were seeded into 6-well plates in DMEM supplemented with 10% FBS and Mn^2+ ^in the absence or presence of differing concentrations of rhFNHN29 and rhFNHC36. After 24 h, cells were incubated in serum-free DMEM for 24 h. The supernatants were collected after centrifugation and the cells were trypsinized and counted. An assay to measure MMP activity was performed using Gelatin Zymography according to Iwai's report [22]. Briefly, a volume of 20 μl of medium was loaded under non-denaturing conditions into a zymogram gel supplemented with 0.1% gelatin to detect the presence of MMP-2 and -9. Electrophoresis was performed at a constant voltage of 120 V for 90 min. Gels were washed in a re-naturing buffer and incubated in an incubation buffer at 37°C for 24 h, stained with Coomassie brilliant blue R-250 (Sigma), and then de-stained with gel-clear de-stain solution. Areas of gelatinolytic degradation appeared as transparent bands on the blue background. The enzymolysis strip volume [area × (gray stripe-gray background)] is expressed as the mean ± SD of the triplicate experiment for each group.

### Western Blot Analysis of Integrin Protein

The MHCC97H cells were cultured as per the process described in "MMP activity". 50 μg samples of cell extract were run on 10% SDS-PAGE gels and electro-blotted to NC membranes, which were then visualized by anti-integrin αv, β3 and β1 mAb, followed by HRP-labeled antimouse IgG and detected by enhanced chemiluminescence (ECL). The relative amount of each protein band was quantified as a ratio to the β-actin band indicated underneath each gel using the densitometric scanning software Quantity One (BIO-RAD). The data are expressed as the mean ± SD of the three independent experiments.

### Protein Tyrosine Phosphorylation

MHCC97H cells were incubated in serum-free DMEM for 10 h, then in DMEM with or without phenylarsine oxide (PAO, 20 μM) for 5 min, and further in DMEM with Mn^2+ ^(0.1 mM) in the presence or absence of FN, rhFNHN29 and rhFNHC36 (200 μg/ml) at 37°C for 20 min. Cell nuclear proteins were extracted and the phosphotyrosyl proteins (p125^FAK^) electroblotted to PVDF membrane were visualized by anti-p-FAK (Tyr 397) after SDS-PAGE, followed by HRP-labeled antirabbit IgG and were detected by ECL.

### Electrophoretic Mobility Band-Shift Assay (EMSA)

The MHCC97H cells were cultured as per the process described in "Protein tyrosine phosphorylation". According to EMSA Kit (Viagene, USA) manufacturer instructions, 7 μg of nuclear proteins were extracted and incubated with the biotin-labeled activator protein 1 (AP-1) doublestranded oligonucleotides probe (5'-CGCTTGATGACTCAGCCGGAA-3'/3'-GCGAACTACTGAGTCGGCCTT-5') in a binding buffer for 20 min at room temperature, and the activated AP-1 was then electroblotted to a nylon membrane and visualized after 6.5% PAGE.

### Statistical Analysis

Results are shown as the mean ± SD. One-way ANOVA (analysis of variance) followed by Fisher's Least Significant Difference post hoc test or Tamhane's T2 test (if equal variance is not assumed) was used for comparison of data from different groups. Differences with *P *< 0.05 were considered statistically significant. For the counting of the lung metastatic foci, the Intraclass Correlation Coefficient (ICC) value was used to evaluate the inter-observer variability (reproducibility) by the reliability analysis of SPSS (Statistical Product and Service Solutions).

## Results

### Purification and Heparin Affinity of rhFNHN29 and rhFNHC36 Polypeptides

**Figure 1:**
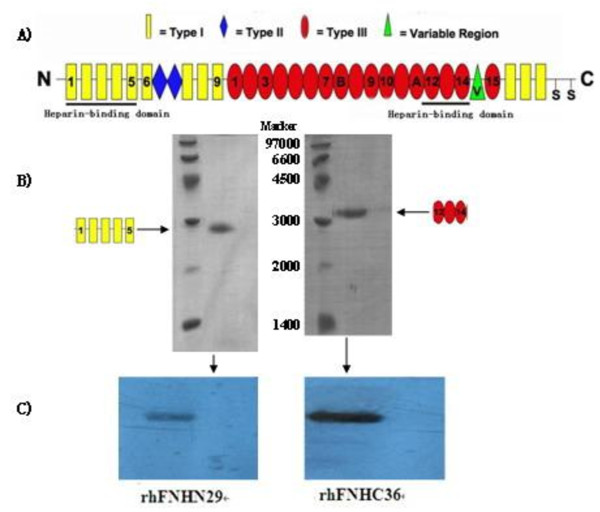
**Expression of purified rhFNHN29 and rhFNHC36 polypeptides**. (A), Schematic structure of FN. FN is made up of a series of repeating homology units of three different types (FNI, FNII and FNIII). Yellow rectangles represent FNI modules, blue rhombus represent FNII modules and red ovals represent specific FNIII modules. RhFNHN29 containing N-terminal heparin-binding domain (HBD) is comprised of the FNI 1-5 modules, and rhFNHC36 containing C-terminal HBD is comprised of the FNIII12-14 modules. (B), Purified rhFNHN29 and rhFNHC36 polypeptides were subjected to SDS-PAGE and visualized by Coomassie Blue staining. (C), The purified rhFNHN29 and rhFNHC36 polypeptides were analysed by Western blot. Immunoreactive fragments of 27.9 kDa and 31.0 kDa indicated their antigenicity.

The N-terminal heparin-binding domain polypeptide of FN consists of five I homologous structures (Figure 1A), containing 237 amino acids (Ser46-Gly282). The length of the DNA sequence encoding this polypeptide is 711 bp (403 bp-1113 bp). In this study, the PCR amplified fragment was 741 bp, and the double-digested fragment was 729 bp. The C-terminal heparin-binding domain polypeptide of FN consists of three III homologous structures (III-12, III-13 and III-14) (Figure 1A), containing 272 amino acids (Tyr1720-Tyr1991). The length of the DNA sequence encoding this polypeptide is 816 bp (5428 bp-6244 bp). In this study, the PCR amplified fragment was 835 bp, and the double-digested fragment was 828 bp. We named the two polypeptides rhFNHN29 and rhFNHC36, respectively.

**Figure 2:**
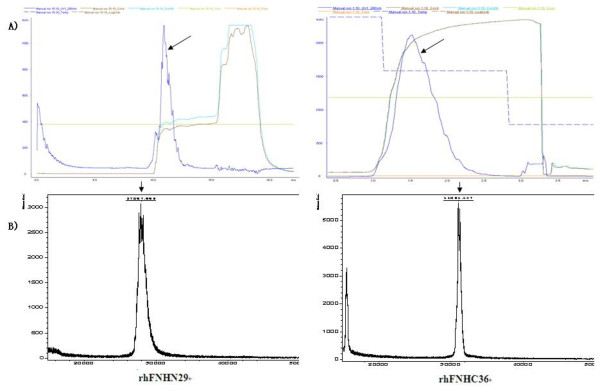
**Heparin affinity analysis of rhFNHN29 and rhFNHC36 polypeptides**. (A), The chromatography showed the peak fractions (Arrow) of eluted rhFNHN29 and rhFNHC36 polypeptides on a column (1 ml) of HiTrap Heparin HP at a flow rate of 1.5 ml/min. (B), LC/MS analysis indicated the elution position of molecular weight for purified rhFNHN29 (27.897 kDa) and rhFNHC36 polypeptides (31.051 kDa).

Upon obtaining yeast expression vectors pPIC9K-FNHN29 and pPIC9K-FNHC36, we successfully established yeast strains GS115-FNHN29 and KM71-FNHC36 that exhibited high expression. The purity of rhFNHN29 and rhFNHC36 polypeptides, which were purified from fermentation supernatant through centrifugalization, ultrafiltration, ion exchange chromatography, and sieve chromatography (Figure 1B), was over 95%. Western blot confirmed the detection of rhFNHN29 and rhFNHC36 with FN polyclonal antibody (Figure 1C). Heparin affinity chromatography indicated that rhFNHN29 and rhFNHC36 display the activity of binding heparin (Figure 2A). Mass spectrometry analysis provided evidence that the molecular weights were 27.9 kDa and 31.0 kDa, respectively (Figure 2B).

### Expression Pattern of Integrins and MMPs in Human Liver Cancer Cell Lines

**Figure 3:**
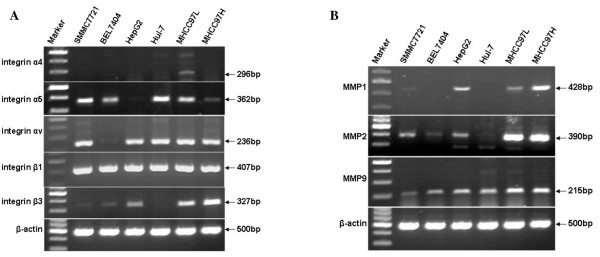
**Standard RT-PCR analysis of different integrins or MMP expression in human liver cancer cell lines**. Total RNA was isolated from human liver cancer cell lines and subsequently reverse-transcribed and analysed by PCR amplification for expression of integrin α4, α5, αv, β1 and β3 (***A***), MMP1, MMP2 and MMP9 (***B***) and, as internal control, β-actin (lower panel). Cell lines are as follows: SMMC7721, BEL7404, HepG2, Huh-7, MHCC97L and MHCC97H.

Some previous studies have demonstrated that different expression levels of integrins and matrix metalloproteinases (MMPs) exhibit different abilities in adhesion and invasion of tumor cells that in turn influence the interaction between the cells and extracellular matrix [23,24]. However, little is known regarding integrin and MMP expression patterns in liver cancer. We are the first to investigate the potential impact of rhFNHN29 and rhFNHC36 on the adhesion and invasion of liver cancer cells by comparing the mRNA expression level of integrins and MMPs in MHCC97H cells (highly invasive) with the expression level found in other human liver cancer cell lines using RT-PCR. The results showed no mRNA expression of integrin α4, low expression levels of integrin α5 mRNA and high levels of integrin αv, integrin β1, integrin β3, MMP1, MMP2 and MMP9 mRNA expression in the MHCC97H cell line (Figure 3). Overall, the MHCC97H cell line appears to be a suitable cell model in order to evaluate the therapeutic potential for rhFNHN29 and rhFNHC36 on invasion and metastasis of liver cancer cells.

### RhFNHN29 and RhFNHC36 Inhibit MHCC7H Cell Adhesion and Migration Induced by Integrin αvβ3

**Figure 4:**
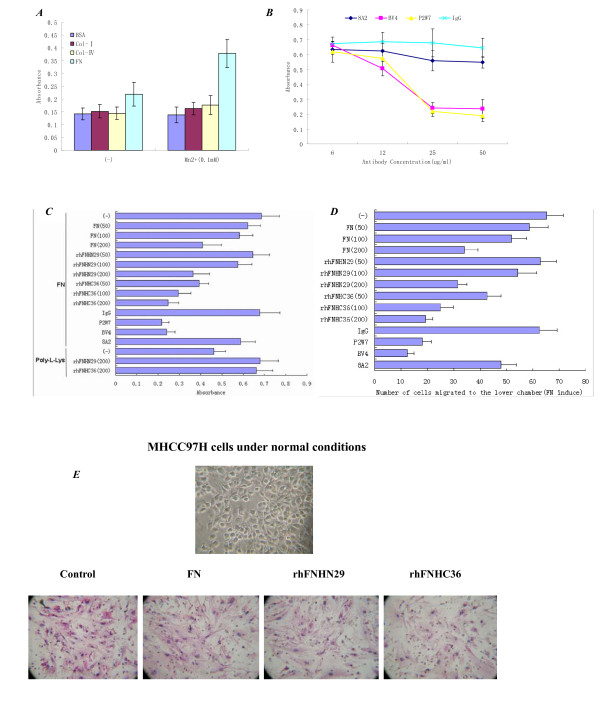
**RhFNHN29 and rhFNHC36 inhibit MHCC97H cell adhesion to FN substrate and migration toward FN**. (A), MHCC97H cell suspension (2 × 10^5^/100 μl) with or without Mn^2+ ^(0.1 mM) was added to a 96-well plate coated with either BSA, type I collagen (20 μg/ml), type IV collagen (20 μg/ml) or FN (20 μg/ml). (B), Effects of different antibody concentrations (6, 12, 25 and 50 μg/ml) on MHCC97H cell adhesion to FN. (C), MHCC97H cell suspension (2 × 10^5^/100 μl) containing Mn^2+ ^(0.1 mM) was pretreated with or without 25 μg/ml control IgG and function-blocking mAb to αv (P2W7), β3 (BV4) or β1 (8A2), respectively, and then seeded to a 96-well plate coated with FN (20 μg/ml) or poly-L-Lys (20 μg/ml) in the presence or absence of FN, rhFNHN29 and rhFNHC36 (50, 100 and 200 μg/ml), respectively. The data represent the mean ± SD of three determinations. (D), A cell invasion assay was carried out in blind-well chambers. MHCC97H cell suspension (5 × 10^4^/100 μl) containing Mn^2+ ^(0.1 mM) treated with or without FN, rhFNHN29 and rhFNHC36 (50, 100 and 200 μg/ml), and control IgG, or anti-αv (P2W7), -β3 (BV4) or -β1 (8A2) (25 μg/ml) were incubated at 37°C for 1 h and then added to the top chambers, respectively. After incubation for 48 h, the number of cells migrating across the Matrigel-coated filter into the bottom chamber was counted. The data represent the mean ± SD of three determinations. (E), Compared with the morphous of MHCC97H cells under normal conditions (×200 magnification), representative pictures for migrated MHCC97H cells on the bottom chamber (×100 magnification) in the presence of FN, rhFNHN29 and rhFNHC36 (200 μg/ml) showed a fibroblastic appearance.

Based on the results above, we further assessed the anti-adhesive effects of rhFNHN29 and rhFNHC36 on MHCC97H cells. As shown in Figure 4A, MHCC97H cells adhered well to the FN substrate, but adherence was dependent on Mn^2+ ^and was affected slightly by BSA and types I and IV collagen substrates. On the other hand, as shown in Figure 4B, we carried out an adhesion inhibition experiment using anti-αv (P2W7) and anti-β3 (BV4) mAb at different concentrations (6, 12, 25 and 50 μg/ml) to verify the type of integrin that mediates adhesion of liver cancer cells to FN. The results showed that anti-αv (P2W7) and anti-β3 (BV4) mAb obviously inhibit MHCC97H cell adhesion to FN at 25 μg/ml, while the differences of the effects of anti-β1 (8A2) on inhibition was not obvious at other concentrations. Thus, we chose 25 μg/ml as the antibody concentration for follow-up experiments. As shown in Figure 4C, MHCC97H cell adhesion to the FN substrate was blocked by anti-αv (P2W7) and anti-β3 (BV4) mAb, but not by anti-β1 (8A2) mAb. RhFNHC36 inhibited MHCC97H cell adhesion to the FN substrate in a dose-dependent manner. RhFNHN29 and FN also inhibited adhesion of MHCC97H cells to the FN substrate but required high concentrations. RhFNHN29 and rhFNHC36 did not affect nonspecific adhesion of MHCC97H cells to poly-L-Lys substrate.

When we performed the cell invasion assay using Transwell chambers, as shown in Figure 4D, the MHCC97H cells migrated across the Matrigel-coated filter into the bottom chamber in response to FN, which was prevented by anti-αv (P2W7) and anti-β3 (BV4) mAb; migration was not prevented by anti-β1 (8A2) mAb, indicating that ligation of αvβ3 induced the invasion of MHCC97H cells. RhFNHC36 inhibited the FN-induced, integrin αvβ3-mediated invasion of MHCC97H cells at a lower dose. RhFNHN29 and FN also affected the invasion of MHCC97H cells to the FN substrate but required higher concentrations.

### RhFNHN29 and RhFNHC36 Decrease Experimental Metastasis of MHCC97H Cells

**Figure 5:**
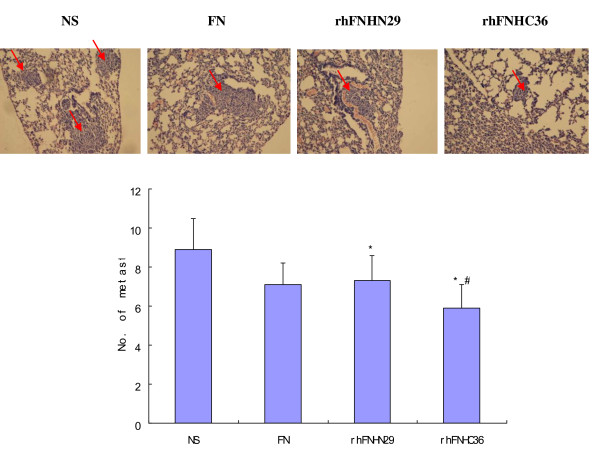
**Effect of rhFNHN29 and rhFNHC36 on metastatic tumor colonies**. Representative pictures of metastasis in lungs of the nude mice 6 wk after MHCC97H cell inoculation and being subjected to intervention by NS, FN, rhFNHN29 and rhFNHC36 (HE staining × 100 →lung metastasis). The group data represent the mean ± SD (n = 8). * denotes a statistically significant difference compared to the control (*P *< 0.05) and # denotes a statistically significant difference with rhFNHN29 (*P *< 0.05) by One-Way ANOVA.

MHCC97H cells exhibit lung metastasis from primary lesions in nude mice [18]. We further investigated the effect of rhFNHN29 and rhFNHC36 on the lung metastasis of MHCC97H cells. Six weeks after MHCC97H cells were inoculated subcutaneously into nude mice, metastasis in the lungs was carefully observed on serial microscopic sections of whole specimens in all mice. As shown in Figure 5, FN, rhFNHN29 and rhFNHC36 intervention groups resulted in a mean number of tumor colonies of 7.1 ± 1.1, 7.3 ± 1.3, and 5.9 ± 1.2, respectively. In contrast, the mean number of lung tumor colonies in the NS group was 8.9 ± 1.6. The lung metastasis of MHCC97H cells was inhibited significantly in nude mice by rhFNHN29 and rhFNHC36 (*P *< 0.05), and the inhibition of rhFNHC36 was more effective than that of rhFNHN29 (*P *< 0.05). Our data suggested that rhFNHN29 and rhFNHC36 might play a role in reducing migration and invasion of MHCC97H cells.

### RhFNHN29 and RhFNHC36 Reduce Integrin Expression and MMP Activity

**Figure 6:**
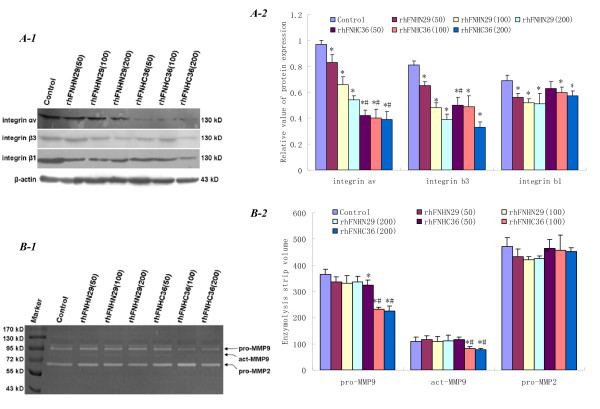
**Reduction of integrin expression and MMP activity by rhFNHN29 and rhFNHC36**. (A-1), Representative immunoblots for integrin αv, β3 and β1 proteins. (A-2), The group data represents the mean ± SD (n = 3). The densitometry data were normalized to β-actin. * denotes a statistically significant difference compared to the control (*P *< 0.05) and # denotes a statistically significant difference compared to rhFNHN29 at the same concentration (*P *< 0.05) by One-Way ANOVA. (B-1), Analysis of MMP activity. Supernatants from MHCC97H cells cultured with Mn^2+ ^in the absence or presence of different concentrations of rhFNHN29 and rhFNHC36 were performed by Gelatin Zymography. (B-2), The group data of enzymolysis strip volume represent mean ± SD (n = 3). * denotes a statistically significant difference compared to the control (*P *< 0.05) and # denotes a statistically significant difference compared to rhFNHN29 at the same concentration (*P *< 0.05) by One-Way ANOVA.

To verify whether the changes in integrin and MMP expression contributed to the reduction of adhesion and invasion of MHCC97H cells by intervention of rhFNHN29 and rhFNHC36, we evaluated the expression of related integrins and activity of MMP-2 and MMP-9 in MHCC97H cells. As shown in Figure 6A, rhFNHN29 and rhFNHC36 affected the expression of integrin αv, β3 and β1 in a dose-dependent manner. Furthermore, integrin αv and β3 expression were almost lost as the cells were treated with rhFNHC36 at a high concentration (200 μg/ml). This indicated that the effectiveness of rhFNHC36 was stronger than that of rhFNHN29. In addition, the impact of rhFNHN29 and rhFNHC36 on activity of proMMP-2 was not significant, but the impact of rhFNHC36 on activity of actMMP-9 and proMMP-9 was significant with concentration-dependence (Figure 6B). Taken together, these results account for the decrease of adhesion and invasion of MHCC97H cells.

### Inhibition of Adhesion and Migration by RhFNHN29 and RhFNHC36 Correlates with Tyrosine Phosphorylation of FAK and Activation of AP-1

**Figure 7:**
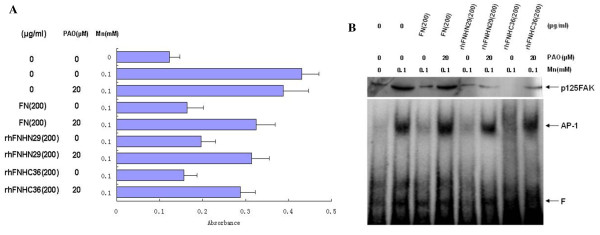
**Effects of rhFNHN29 and rhFNHC36 on tyrosine phosphorylation of FAK and activation of AP-1**. (A), Effect of PAO on the anti-adhesive activity of rhFNHN29 and rhFNHC36. MHCC97H cell suspension (2 × 10^5^/100 μl) with or without Mn^2+^(0.1 mM), with or without PAO (20 μM) were pretreated with FN, rhFNHN29 and rhFNHC36 (200 μg/ml), respectively, and then seeded in a 96-well plate coated with or without FN (2.5 μg/ml). Adhesion of MHCC97H cells to the FN substrate was assayed under the indicated conditions as described in Figure 2. (B), Immunoblotting of p125^FAK ^and EMSA of activated AP-1. MHCC97H cells were deprived of serum for 10 h, incubated in DMEM with or without PAO (20 μM) for 5 min, and further cultured with Mn^2+^(0.1 mM) in the presence or absence of FN, rhFNHN29 and rhFNHC36 (200 μg/ml) at 37°C for 20 min. The cytoplasmic protein and the nuclear protein were extracted respectively. The phosphotyrosyl proteins electroblotted to the PVDF membrane were visualized by anti-p-FAK (Tyr 397) after SDS-PAGE, and the activated AP-1 electroblotted to Nylon membrane was visualized by specific HRP-labeled probe after 6.5% PAGE for nuclear protein (*F *zone indicates non-binded free probe).

Protein tyrosine phosphorylation of FAK is considered to play a crucial role in integrin-mediated signaling events. To further elucidate the molecular mechanism and investigate the extended integrin specificity of rhFNHN29 and rhFNHC36, we examined the effect of phenylarsine oxide (PAO), a protein tyrosine phosphatase inhibitor [25], on the anti-adhesive activity of rhFNHN29 and rhFNHC36. As shown in Figure 7A, the inhibitory effect of rhFNHN29 and rhFNHC36 on MHCC97H cell adhesion to the FN substrate was reversed by the addition of PAO, although PAO itself had no effect on MHCC97H cell adhesion. Next, we observed tyrosine phosphorylation of FAK (p125^FAK^) and activation of AP-1 involved in adhesion and migration signaling. When MHCC97H cells were stimulated in the presence of Mn^2+^, rhFNHN29 and rhFNHC36, p125^FAK ^and activated AP-1 remained only at basal levels. The decreased p125^FAK ^and AP-1 were restored by the addition of PAO (Figure 7B).

## Discussion

As the recurrence and metastasis of HCC remains a formidable problem in clinical practice, many efforts have been made to develop a more efficient treatment to inhibit or prevent tumor metastasis. As the expression level of integrins on HCC cells and in the extracellular matrix is related closely to a cell's ability to migrate [26,27], attempts to realize "anti-adhesion therapy" have been undertaken using synthetic integrin-binding peptides derived from cell adhesive sites of ECM protein. A number of previous studies have proposed that synthetic peptides which contain the RGD cell adhesion motif common in several ECM proteins, including FN, have the potential to prevent tumor metastasis [28]. However, the application of these short peptides is limited due to high dosage requirement and short half-life. To overcome these difficulties, various chemical modifications of the integrin-binding peptides have been performed in order to enhance integrin binding affinity [29,30]. A synthetic peptide derived from the 14th type III module weakens the integrin α5β1-mediated adhesion of various cell types to the FN substrate in a reversible manner [14]. Wang [31] designed a derivative of synthesized peptides containing anti-adhesion peptide β (DLYYLMDLSYSMK), in accordance with the conserved sequence of the integrin α and β unit. Liu [13] demonstrated that treatment with the gene delivery technique resulting in *in vivo *expression of a recombinant CBD-HepII bifunctional-domain polypeptide of FN, designated as CH50, inhibited tumor metastasis to the liver. However, the impact of this gene therapy on liver cells themselves has not been investigated.

The present study demonstrated that the polypeptides derived from FN, rhFNHN29 and rhFNHC36, have the ability to reduce by ~30% the number of lung metastases of MHCC97H cells in the nude mice metastasis model. This efficacy might be explained as less degradation with longer fragment and less of an effect on the model organism with non-chemical modification and non-gene expression in cells. The mechanisms underlying the inhibition may be very complex, yet they were identified in this study as follows.

First of all, rhFNHN29 and rhFNHC36 could interfere with the function of integrin αv and β3. As to the αvβ3-mediated interaction, rhFNHN29 and rhFNHC36 inhibited the MHCC97H cell migration toward the FN concentration gradient, as well as adhesion to the FN substrate. Previous studies have confirmed that human recombinant fibronectin polypeptide CH50 has the ability to interfere with the expression and activity of integrin αv and β3, resulting in the inhibition of invasive growth of tumor cells and angiogenesis [13], and a 22-mer FN peptide, termed FNIII14, has also been shown to inhibit β1 integrin-mediated adhesion without binding to integrins and has exhibited the potential to prevent lymphoma cell metastasis [14]. It is understood that no basement membranes exist under liver sinusoidal endothelial cells, thus liver cancer cells can not have the same adhesion to laminin or collagen as other tumor cells, and so it follows that integrin-mediated HCC metastasis and cell adhesion to FN play a decisive role in the formation of metastases [32-34]. This involves several types of FN receptors, such as α4, α5, αv and β1, β3 integrin subunit, of which integrin αvβ3 is the key element that mediates liver cancer invasion and metastasis due to low expression in normal tissue but high expression in HCC [34]. Therefore, intervention with FN-type peptides is more suitable for liver cancer cells that express α4, α5, αv, β1 or β3 integrin.

The Integrin-FAK-AP-1 signaling pathway plays an important role in cell adhesion, proliferation, migration, differentiation, etc. [35]. Intracellular signaling involves the accumulation of several proteins such as focal adhesion kinase (FAK), which is mainly distributed in the adhesion plaque (FAP) structure. After the extracellular domain of integrin α subunit contacts the ECM, the integrins on the membrane cluster and further activate adhesion kinase (FAK phosphorylation) and other protein kinases [36,37]. The nuclear target proteins, including AP-1 transcription factor, receive the activated signal and further control gene expression. During the adhesion and invasion process of hepatocellular carcinoma cells, the tyrosine kinase-mediated signaling pathways induced by integrin receptors are of great importance [38]. In this study, it was found that rhFNHN29 and rhFNHC36 inhibit MHCC97H cell adhesion and invasion by blocking the integrin-FAK-AP-1 pathway. Expression of p-FAK was exhibited when a tyrosine phosphatase inhibitor (PAO) was added, suggesting that the interaction between rhFNHN29 or rhFNHN36 and integrin alpha V beta 3 results in the blockage of a series of signals into the nucleus including the AP-1 activation signal. The promoters regulated by AP-1 include TRE of integrins [39] and MMPs [40], which contain AP-1 binding sites, and TPA response elements [41]. It was also found that the inhibitory impact of rhFNHC36 on integrin and MMP9 expression exhibited dose-dependence, but impact of rhFNHN29 on integrin expression required a high concentration. The rhFNHC36 polypeptide is more effective than rhFNHN29 due to the following: 1) although both rhFNHN29 and rhFNHC36 contain a heparin-binding domain, rhFNHC36 contains another anti-adhesion site, YTIYVIAL, which was found by Kato [14]. This anti-adhesive site is buried within the type III module structure in plasma FN, however, it is exposed in rhFNHC36. 2) AP-1 transcription factors are leucine zipper proteins that bind to a consensus DNA sequence (5'-TGAG/CTCA-3') as a dimeric complex. Various AP-1 dimers bind to DNA with differing affinities. The dimers of AP-1 bound with MMP9 promoter DNA are greater than that of MMP2 [40,42], hence regulation of AP-1 due to MMP9 expression is more sensitive than through MMP2 expression. We speculated that through the integrin-FAK-AP-1 pathway the effect of rhFNHC36 on MMP9 activity by AP-1 may be greater than the effect of rhFNHN29 in MHCC97H cells.

In this study our intervention is targeted. Different tumor cells express different integrins and MMPs, which are often associated with a different potential for tumor invasion and metastasis. In previous reports, mRNA expression levels of integrins and MMPs have been analysed using a panel of human tumor cell lines with different degrees of differentiation and potential for tumor formation, tissue invasion, and metastasis [23,24]. In this study, the mRNA expression of integrins and MMPs that was detected in different liver cancer cell lines and MHCC97H cells was found to feature low levels of integrin α5 mRNA expression and high levels of integrin αv, integrin β1, integrin β3, MMP1, MMP2 and MMP9 mRNA expression. This may partly contribute to its invasion and metastasis, indicating that MHCC97H cells can be used as a model for the study of anti-invasion and anti-metastasis.

In summary, our work provides the first evidence that rhFNHN29 and rhFNHC36 may play an important role in safeguarding against human liver cancer through the reduction of integrin expression level and MMP-9 activity and may shed light on a novel strategy for liver cancer therapy.

## Conclusions

We found that both rhFNHN29 and rhFNHC36 polypeptides were able to inhibit the adhesion and invasion of MHCC97H cells, and moreover abrogate the tyrosine phosphorylation of focal adhesion kinase (p-FAK) and activation of activator protein 1 (AP-1), which resulted in the decrease of integrin αv, β3 and β1 expression and the reduction of MMP-9 activity. These findings suggest that rhFNHN29 and rhFNHC36 may play an important role in controlling human liver cancer invasion.

## Abbreviations

FN: fibronectin; rhFNHN29 and rhFNHC36: N-terminal and C-terminal heparin-binding domain peptides of FN; HCC: hepatocellular carcinoma; ECM: extracellular matrix; FAK: focal adhesion kinase; PAO: phenylarsine oxide; NS: Normal saline; EMSA: electrophoretic mobility band-shift assays

## Competing interests

The authors declare that they have no competing interests.

## Authors' contributions

YZC developed the study concept, aims and initiated the project. NHT, XQW and XJL performed the experimental work described in the study and were responsible for the drafting of the manuscript. YLC participated in the design of this study. YW and QLZ provided valuable scientific suggestions. All authors have read and approved the final manuscript.

## Pre-publication history

The pre-publication history for this paper can be accessed here:

http://www.biomedcentral.com/1471-2407/10/552/prepub
